# Pancreatic Neuroendocrine Tumors in MEN1 Patients: Difference in Post-Operative Complications and Tumor Progression between Major and Minimal Pancreatic Surgeries

**DOI:** 10.3390/cancers15204919

**Published:** 2023-10-10

**Authors:** Francesco Tonelli, Francesca Marini, Francesca Giusti, Teresa Iantomasi, Francesco Giudici, Maria Luisa Brandi

**Affiliations:** 1 Fondazione F.I.R.M.O. Onlus, Fondazione Italiana per la Ricerca sulle Malattie dell’Osso (Italian Foundation for the Research on Bone Diseases), 50129 Florence, Italy; francesco.tonelli@unifi.it (F.T.);; 2 Donatello Bone Clinic, Villa Donatello Hospital, 50019 Sesto Fiorentino, Italy; 3 Department of Experimental and Clinical Biomedical Sciences, University of Florence, 50139 Florence, Italy; 4 Department of Clinical and Experimental Medicine, Surgical Unit, University of Florence, 50139 Florence, Italy

**Keywords:** pancreatic neuroendocrine neoplasms (PNENs), multiple endocrine neoplasia type 1 (MEN1), pancreaticoduodenectomy (PD), distal pancreatectomy (DP), minimal pancreatic surgeries

## Abstract

**Simple Summary:**

We described post-operative complications, pancreatic sequelae, and post-surgical progression/relapse of pancreatic neuroendocrine neoplasms (PNENs) in 43 patients with multiple endocrine neoplasia type 1 (MEN1), who underwent major or minimal pancreatic surgeries. The performance of a more conservative intervention, compared to both pancreaticoduodenectomy (PD) and distal pancreatectomy (DP), was associated with the absence of post-surgical complications, but required further pancreas surgeries for tumor relapse in 40.0% of the operated cases. Our data showed that major pancreatic resections (PD and DP) were effective in both preventing tumoral progression and guaranteeing a long-term PNEN relapse-free survival time in most of N+ and/or M+ patients.

**Abstract:**

Pancreatic neuroendocrine neoplasms (PNENs) affect over 80% of patients with multiple endocrine neoplasia type 1 (MEN1). Surgery is usually the therapy of choice, but the real immediate and long-term therapeutic benefit of a partial extensive pancreatic resection remains controversial. We analyzed, in 43 PNEN MEN1 patients who underwent 19 pancreaticoduodenectomies (PD), 19 distal pancreatectomies (DP), and 5 minimal pancreatectomies, the prevalence of surgery-derived early complications and post-operative pancreatic sequelae, and the PNEN relapse-free survival time after surgery, comparing major (PD+DP) and minimal pancreatic surgeries. No post-operative mortality was observed. Metastatic cancers were found in 12 cases, prevalently from duodenal gastrinoma. Long-term cure of endocrine syndromes, by the 38 major pancreatic resections, was obtained in 78.9% of gastrinomas and 92.9% of insulinomas. In only one patient, hepatic metastases, due to gastrinoma, progressed to death. Out of the 38 major surgeries, only one patient was reoperated for the growth of a new PNEN in the remnant pancreas. No functioning PNEN persistence was reported in the five minimal pancreatic surgeries, PNEN relapse occurred in 60% of patients, and 40% of cases needed further pancreatic resection for tumor recurrence. No significant difference in PNEN relapse-free survival time after surgery was found between major and minimal pancreatic surgeries.

## 1. Introduction

Pancreatic neuroendocrine neoplasms (PNENs) and/or duodenal gastrinomas are found in over 80% of patients with multiple endocrine neoplasia type 1 (MEN1) syndrome. PNENs can be distinguished in functioning (those that are accompanied by increased hormonal production and related endocrine syndrome) and non-functioning tumors (producing no detectable active hormones or secreting pancreatic polypeptide). Pancreatic or duodenal neuroendocrine tumors are discovered when a related endocrine syndrome occurs, or during abdominal examination performed for surveillance of MEN1 patients and asymptomatic carriers of *MEN1* gene mutation. Despite the diagnostic progress, allowing for earlier diagnosis, and the definition of criteria to indicate surgical excision, duodeno-pancreatic neuroendocrine neoplasms (DP-NENs) continue to be the leading cause of MEN1-related death at around 55–60 years of age [1,2,3,4,5,6]. 

Surgery is the gold standard to prevent the malignant progression of DP-NENs in MEN1 patients, and to solve hormone-derived endocrine syndromes. However, the appropriate timing and extent of surgery still remain a matter of debate. Indication to surgery is substantially based on the presence of an endocrine syndrome refractory to medical treatment, the size of a PNEN being more than 2 cm in diameter, and/or tumor(s) showing fast growth. For PNENs smaller than 2 cm, a well-established consensus to surgery does not exist. 

Regarding the extent of surgery, in recent years, the most frequently adopted surgery has been the distal pancreatectomy (DP), associated with the enucleation of all the demonstrable tumors in the duodenum wall and/or in the pancreatic head (Thompson’s procedure). Conversely, pancreaticoduodenectomy (PD) has been performed much more rarely [7,8,9,10,11]. This is certainly due to the complexity of the procedure and the high risk of surgical complications. Both these types of surgery present risk of lifelong post-operative sequelae, the most important of which is diabetes from insufficient insulin production. Moreover, given the genetic nature of the MEN1 syndrome, the residual pancreas and duodenum are at high risk of development of new DP-NENs. Some studies have evaluated the risk of relapse in the residual pancreas after a partial pancreatectomy in MEN1 patients, but the characteristics of the studies were heterogeneous and the post-operative follow-up was generally shorter than 10 years [7,8,9,10,11,12,13,14,15,16,17,18,19]. It is, thus, not clear, from the data currently available in the published literature, if the extensive partial resection of part of the pancreas has the potential to reduce malignant tumor progression and/or prevent the onset of new tumors in the residual gland, particularly those of malignant form.

Here, we analyzed surgical results in 43 Italian MEN1 patients with PNENs, collected from the “Florentine MEN1 database”, who underwent 38 major partial pancreatic resections (19 DPs and 19 PDs) and 5 minimal pancreatic surgeries.

We reported results of post-operative morbidity, curability of functioning PNEN (F-PNEN)-derived endocrine syndromes, oncological survival, and PNEN relapse-free survival time, following different types of surgery, and we specifically compared these post-operative outcomes between major partial pancreatic resections and minimal pancreatic surgeries.

## 2. Materials and Methods

### 2.1. Patients

This retrospective observational study was performed on a group of Italian MEN1 patients retrieved from the “Florentine MEN1 database” [20], as a part of the “Italian MEN1 Database” [21]. The study included 43 consecutive MEN1 patients (28 women and 15 men) who underwent a partial pancreatic resection for PNEN(s) between 1993 and 2018. Data about pancreas surgery, removed F-PNEN(s) and/or non-functioning PNEN(s) (NF-PNENs), post-operative early complications and pancreatic sequelae, post-operative medical therapy for PNENs, the persistence and/or relapse of PNEN, and pancreas surgical reinterventions for PNEN persistence/relapse were all retrieved from the MEN1 database.

### 2.2. Criteria for Surgery and Post-Operative Follow-Up

For inclusion in this study, we selected only patients who underwent a partial pancreatic resection as the first surgery for PNEN(s).

The surgical interventions employed in our series were classified as follows:

(A) Nineteen PDs, consisting of the resection of the head (right side) of the pancreas and part of the duodenum for the removal of duodenal gastrinomas, and cholecystectomy, performed, in the majority of patients, with the pylorus-preserving modality or, alternatively, with reconstruction according to the Whipple procedure. In some patients, the pancreatic resection was extended beyond the pancreatic isthmus, towards the pancreas body, to remove any PNEN ≥ 1cm
(B) Nineteen DPs, consisting of the resection of both body and tail (left side) of the pancreas, usually with a spleen-preserving modality, associated with enucleation(s) of PNENs larger than 1 cm in the pancreas head and/or of duodenal gastrinomas from the duodenal wall (Thompson procedure).
(C) Two minimal resections of the pancreas tail
(D) Three single enucleations of PNENs ≥ 1 cm

PD + DP were gathered into “major partial pancreatic resections”, while resections of the pancreas tail and enucleations were classified together as “minimal pancreatic surgeries”.

In 11 patients submitted to major pancreatic resections, and in the 2 patients who underwent minimal pancreas resection, the enucleation of one or more PNENs in the preserved pancreas was associated. The resection of peripancreatic and celiac lymph nodes was associated with PD, while the removal of splenic lymph nodes was performed in DP. In the presence of hepatic metastases, a radical liver resection was performed.

In our MEN1 patients, indications for pancreatic surgery were the presence of a symptomatic F-PNEN with an endocrine syndrome, such as Zollinger–Ellison syndrome (ZES), insulinoma-caused hypoglycemic crises, or vasoactive intestinal peptide (VIP)-associated syndrome, and/or the presence of a least one NF-PNEN ≥ 2.0 cm.

Follow-up time was calculated from the date of pancreatic surgery to the date of the last check-up reported in the database. Patients included in this study had a mean time of post-operative follow-up of 12.8 ± 7.1 years (range 2–26 years, median 12.0 years). Post-operative follow-up consisted both of biochemical serum dosage, every 12 months, of a panel of hormones and peptides secreted by MEN1-associated F-PNENs (gastrin, insulin with an associated fasting glucose dosage, VIP, glucagon, and pancreatic polypeptide (PP)) and of an imaging screening of the abdomen, performed every 12–24 months, using variable combinations of one or more of the following diagnostic techniques: endoscopic ultrasound (EUS), magnetic resonance imaging (MRI), computed tomography (CT) scan with or without contrast medium, **^111^**Indium-octreotide scintigraphy (Octreoscan), and positron emission tomography/computed tomography (PET/CT) with ^68^Gallium-labeled somatostatin analogues (^68^Ga-PET/CT).

A secretin stimulation test was performed after surgery for the assessment of persistence/recurrence of hypergastrinism.

### 2.3. Post-Operative Morbidity: Complications, Sequelae, and PNEN Persistence and Relapse

Post-operative morbidity was analyzed in all the operated patients, through the evaluation of the incidence of specific early and long-term post-operative outcomes.

Post-operative early complications were considered those that manifested during the hospital stay and up to 30 days after surgery. Data were retrieved on the occurrence of post-operative pancreatic fistula (POPF) grade B or C, post-pancreatectomy hemorrhage (PPH), and post-pancreatectomy acute pancreatitis (PPAP). Only complications classified as Clavien–Dindo grades III (requiring surgical, endoscopic, or radiological intervention) and IV (life-threatening complication requiring an intensive care unit) were evaluated in this study.

Post-operative mortality was defined as a death within 30 days after surgery.

As long-term post-operative outcomes, we considered two pancreatic sequelae: new-onset diabetes mellitus (DM) and exocrine pancreatic insufficiency (EPI). The occurrence of post-operative DM was defined when the operated patient required the use of antidiabetic medication for 6 months or more after surgery. The occurrence of post-operative chronic EPI was defined when the operated patient required the use of pancreatic enzyme supplementation for 6 months or more after surgery.

As indicators (primary outcomes) of tumor progression, we evaluated:

1. The persistence of endocrine syndrome caused by F-PNENs. Disease persistence was defined in the presence of hormone over-secretion found up to 3 months after surgery. Patients with elevated gastrin and/or a positive secretin stimulation test were considered to have a persistent gastrinoma, while patients with hypoglycemia (confirmed also via a fasting blood sugar test) were considered to have a persistent insulinoma.
2. The recurrence of lymph node metastases or distant metastases identified more than 3 months after surgery.
3. The relapse of F-PNENs and/or NF-PNENs, due to newly developed primary tumor(s) in the remnant endocrine pancreas, identified more than 3 months after surgery through at least one of the imaging screenings of the abdomen.

The PNEN relapse-free survival time after surgery was calculated from the date of pancreatic surgery to the date of the first post-operative diagnosis of PNEN relapse.

### 2.4. Statistical Analysis

Continuous variables were calculated as mean values ± standard deviation (SD) and/or median with the range of values, while categorial variables were counted as percentages. Statistical comparisons between the two groups of patients treated with major or minimal pancreatic surgeries were performed using the non-parametric Student’s *t*-test for continuous variables or the Fisher’s exact test for categorial variables, assuming, for all tests, a *p* value less than 0.05 as an indicator of statistical significance (over 95%).

Kaplan–Meier analysis was performed to estimate the cumulative PNEN relapse-free survival after surgery, through computing the fraction of patients who were free from PNEN relapse at each time point. The log-rank test was used to compare PNEN relapse-free survival after a major partial pancreatic resection or a minimal pancreatic surgery.

## 3. Results

### 3.1. Patients and Surgery

This study included 43 patients who were clinically and/or genetically diagnosed with MEN1 at a mean age of 30.9 ± 10.1 years (median 29.0; range 15–57 years). They had a diagnosis of PNEN at a mean age of 35.0 ± 11.1 years (median 34.0; range 17–63 years). They all underwent a partial pancreatic resection for PNEN(s), with a mean age at surgery of 36.4 ± 11.5 years (median 35.0; range 17–63 years), and with a mean time of 1.4 ± 2.8 years (median 1.0; range 0–18 years) occurring between the diagnosis of PNEN and the surgical intervention.

Nineteen patients (44.2%) underwent a PD, and nineteen patients (44.2%) underwent a DP, while five patients (11.6%) underwent minimal pancreatic surgeries, consisting of two single enucleations in the pancreas head (4.7%), one single enucleation in the pancreas tail (2.3%), and two pancreas tail resections (4.7%), one of them associated with a single enucleation in the pancreas head. In addition to these 43 cases, in our MEN1 database, we reported only one patient who required a total pancreatectomy as a first surgical intervention for metastatic multiple gastrinomas and 25 NF-PNENs scattered on the entire pancreas, which could not be removed with a partial resection of the organ.

Surgery indications for the 19 PDs were the presence of ZES in 12 cases (63.2%), insulinoma with hypoglycemic crises in 3 cases (15.8%), VIP syndrome in 1 case (5.3%), and the presence of at least one NF-PNEN ≥ 2.5 cm in 3 cases (15.8%).

Surgery indications for the 19 DPs were insulinoma with hypoglycemic crises in 10 cases (52.6%), the presence of at least one NF-PNEN ≥ 2.0 cm in 7 cases (36.8%), and the presence of ZES in 2 cases (10.5%).

Surgery indications for the five minimal pancreatic surgeries were insulinoma with hypoglycemic crises in three cases (60.0%) and the presence of ZES in two cases (40.0%).

Out of the 19 PDs, 7 (36.8%) were associated with a contemporary resection/enucleation in the pancreas body or tail (3 with resection of the pancreas body, 2 with resection of the pancreas tail, and 2 with enucleation in the pancreas tail). Liver resection was performed in four cases (21.0%) for suspected hepatic metastases. Two liver resections confirmed the presence of hepatic metastases, while in two cases, the removed hepatic lesions resulted to be focal nodular hyperplasia.

Out of the 19 DP, 4 (21.0%) were associated with a contemporary enucleation in the pancreas head, 2 with a duodenal enucleation of single gastrinoma (10.5%), and 5 with the cholecystectomy (26.3%). No liver resection was performed in DP cases.

Two liver resections (40.0%) were performed in the five patients who underwent a minimal pancreatic surgery for suspected hepatic metastases. All these two liver resections resulted to be focal nodular hyperplasia during post-operative histological analyses.

At least one PNEN ≥ 2 cm but less than 3 cm was removed in 17 of the 43 operated cases (39.5%), including eight from the 19 PDs (42.1%), eight from the 19 DPs (42.1%), and one from the 5 minimal pancreatectomies (20.0%), while at least one PNEN ≥ 3 cm was removed in 6 of all the 43 operated patients (14.0%), including two from the 19 PDs (10.5%), three from the 19 DPs (15.8%), and one from the 5 minimal pancreatectomies (20.0%).

Gastrinomas and insulinomas were the two most common resected F-PNENs in our patients. Gastrinomas were removed in 16/19 (84.2%) of PD-operated patients, in 3/19 (15.8%) of DP-operated patients, and in 2 (40.0%) of the 5 patients who underwent a minimal pancreatic surgery. The great majority of gastrinomas were found in the duodenum wall (second and third portions); only three patients had a pancreatic gastrinoma, of which two were in presence of one or more concomitant duodenal gastrinomas. Furthermore, one patient, in addition to duodenal gastrinomas, had a gastrinoma of the gallbladder. In 42.9% of cases, patients with gastrinoma also had gastrinoma-derived metastasis in the peripancreatic lymph nodes, at the time of surgery. 

Insulinoma were removed in 3/19 (15.8%) of PD-operated patients, in 11/19 (57.9%) of DP-operated patients, and in 3 (60.0%) of the patients who underwent a minimal pancreatic surgery.

Metastatic PNENs were resected in 12 of the 19 PD-operated patients (63.2%), of which 10 had only lymph node metastases, one had only liver metastases, and one had both lymph node and liver metastases. No metastatic tumors were resected in all the 19 cases operated via DP and all the 5 patients who underwent a minimal pancreatic surgery. The great majority of metastases were from duodenal gastrinoma and positive to gastrin immunostaining. Insulin-secreting metastases were resected in two cases.

Microscope histological evaluation of the resected tumors showed they were all well-differentiated PNENs. The analysis of the Ki67 index, on both F-NENs and NF-NENs, showed a value of less than 5% for all the analyzed tumors. 

Surgical characteristics in the two groups of patients treated with major or minimal pancreatic surgeries, expressed as the mean ± SD, median, and range of values, or as a percentage, are reported in Table 1. 

### 3.2. Post-Operative Early Complications

Post-operative early complications occurred in 9 out of the 43 operated patients (21.0%); of these, 2 occurred among the 19 PD cases (10.5%) and 7 occurred among the 19 DP cases (36.8%). None of the five patients who underwent a minimal pancreatic surgery showed any post-operative early complication.

In PD cases, we reported:

- One patient with POPF grade C;
- One patient with PPAP.

In the first case, the POPF caused a hemorrhage and abdominal abscess, which required post-operative total pancreatectomy (classified as Clavien–Dindo grade IV).

In DP cases, we reported:

- Two patients with PPAP;
- One patient with PPAP and PPH;
- One patient with PPH and retro-pancreatic hematoma;
- Three patients with a POPF (one grade C and two grade B).

One of the patients with PPAP, the patient with PPAP and PPH and the patient with POPF grade C required a post-operative early reintervention for the treatments of these DP-derived complications (all classified as Clavien–Dindo grade III).

Late post-operative DM occurred in 16 of the 43 operated patients (37.2%), including 10 operated via PD (62.5% of DM cases), 5 via DP (31.3% of DM cases), and 1 via pancreas tail resection associated with enucleation in the pancreas head (6.2% of DM cases).

Post-operative chronic EPI occurred in 23 of the 43 operated patients (53.5%), including 15 operated via PD (65.2% of EPI cases), 7 via DP (30.4% of EPI cases), and 1 via the pancreas tail resection associated with enucleation in the pancreas head (4.3% of EPI cases).

Both of these two pancreas sequelae resulted in needing to be controlled/normalized through pharmacological therapies, in all the affected cases, during the entire period of post-operative follow-up analyzed in this study.

### 3.3. Post-Operative Persistence of Endocrine Syndromes

Post-operative persistence of F-PNEN-derived endocrine syndromes was reported in three cases of PD (15.8%), all due to the persistence of hypergastrinemia and positivity of the secretin stimulation test, caused by residual gastrin-secretin lesions, and in two cases of DP (10.5%), one being persistent hypergastrinism and one being hypoglycemia from an insulin-secreting lesion. 

Out of the four total cases of persistence of hypergastrinism, (1) one was due to an ectopic gastrinoma in the biliary duct, and it was successfully surgically resected 15 months after the PD; (2) two were caused by lymph node metastatic gastrinomas, treated with four cycles of chemotherapy with dacarbazine and 5-fluorouracil, and with anti-acid drugs, respectively; (3) one was presumably due to a residual gastrinoma smaller than 1 cm in the duodenum or in the pancreas head in one patient who underwent DP. This patient was treated with somatostatin analogues (SSAs).

One patient with persistence of insulinoma was pharmacologically treated with diazoxide.

No persistence of endocrine syndromes was reported in all the five cases who underwent a minimal pancreatic surgery.

### 3.4. Post-Operative Relapse of F-PNENs

Five cases of F-PNEN relapse (26.3%) were reported after PD, with a mean time between the PD and the relapse discovery of 5.0 ± 5.2 years (median 3.0; range 1.5–14 years). Out of these five cases, four were caused by extra-pancreatic gastrin-secreting lesions, and one was caused by a newly developed glucagon-secreting PNEN in the pancreas tail.

After DP, we reported only one case of recurrence of a new primary gastrinoma (5.3%) discovered 12 years after the surgery. This patient manifested a contemporary relapse of multiple NF-PNENs, which had arisen in the pancreas head and in the remaining section of the pancreas body.

Only one F-PNEN relapse was reported in one of the five patients who had underwent a minimal pancreatic surgery (20.0%). This patient developed a new primary insulinoma in the body–tail junction of the pancreas, discovered 14 years after the first single enucleation of an insulinoma in the pancreas head, which required a DP, followed by exocrine insufficiency of the pancreas. No further PNEN persistence or relapse was reported during the following 10 years, after this second surgery.

### 3.5. Post-Operative Relapse of NF-PNENs

Five cases of NF-PNEN relapse (26.3%) were reported after PD, with a mean time between the PD and the relapse discovery of 5.3 ± 4.5 years (median 4.3; range 1–10 years). Out of these five cases, four were due to the development of new primary PNENs in the remnant pancreas, and one was caused by newly discovered metastases in two retroperitoneal lymph nodes. Of the four cases of new primary non-functioning tumors within the pancreas, three manifested as a single lesion (one tumor of 0.4 cm, one tumor of 2.3 cm, and one PNEN larger than 1 cm of which the exact dimension is not reported in the database), while the fourth case showed multiple lesions, consisting of three tumors, of which one measured 1.3 cm and two were less than 0.5 cm. Two of these relapsed patients underwent a second pancreas surgery, one consisting of the enucleation of a single NF-PNEN greater than 1 cm in the pancreas body, 16 months after the first PD, and one consisting of a DP (with conservation of part of the pancreas body), for removing three NF-PNENs, one in the pancreas tail of 2.8 cm size and two in the pancreas body of 1.6 and 0.6 cm size, 15 years after the first PD.

Ten cases of NF-PNEN relapse (52.6%) were reported after DP, all due to the development of new primary tumors in the remnant pancreas, with a mean time between DP and tumor relapse discovery of 10.3 ± 7.2 years (median 12.5; range 0.5–20 years). All these ten relapse cases except one manifested at least two newly developed lesions. In three cases, we reported at least one of the new primary tumors having a diameter > 1 cm. None of these ten relapsed patients underwent a second pancreatic surgery during the follow-up period included in this study; five of them (50.0%) were treated with SSA therapy. 

In the five cases who underwent a minimal pancreatic surgery, we reported two cases of NF-PNEN relapse. Of these, one consisted of a single tumor in the pancreas body, identified 1 year after the resection of the pancreas tail, and one consisted of multiple small tumors (more than 5 lesions all ≤ 0.7 cm) in the body–tail region of the pancreas, discovered 6 years after the first single enucleation of one insulinoma in the pancreas tail. The patient with relapse of a single NF-PNEN underwent a second single enucleation in the pancreas body, one year after the first resection of pancreas tail, followed by post-operative pancreatic abscess. Thirteen years after this second pancreatic surgery, this patient needed further reoperation for new PNENs in the pancreas head and multiple metastases in the liver; PD and radiofrequency ablation of liver metastases were performed. After this third reoperation, the patient was treated long-term with SSAs that guaranteed a PNEN recurrence-free period during the further 11 years of available follow-up. The relapsed patient who developed new primary multiple small PNENs was treated with SSAs, which showed to be able to control tumor growth during the 11 years of follow-up between the discovery of these novel PNENs and the last performed imaging evaluation of the abdomen.

Our 43 operated patients showed an average PNEN relapse-free survival time after surgery of 9.3 ± 6.3 years (median 10.0; range 1–25 years). The 19 PD-operated patients had an average PNEN relapse-free time after surgery of 8.7 ± 6.6 years (median 10.0; range 1–25 years); the 19 operated via DP, of 10.2 ± 6.5 years (median 11.0; range 0.5–20 years); and the 5 who underwent a minimal pancreatic surgery, of 8.7 ± 5.3 years (median 9.5; range 1–14 years), with no significant differences between the three groups of surgeries.

**Figure 1:**
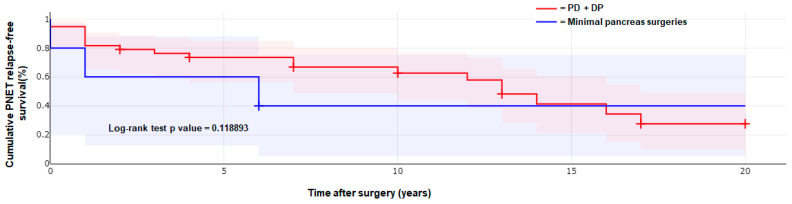
Kaplan–Meier plotted curves showing PNEN relapse-free survival in the 38 patients who underwent pancreaticoduodenectomy (PD) or distal pancreatectomy (red line) and in the 5 patients who underwent minimal pancreas surgery (blue line).

Kaplan–Meier analysis was performed to estimate and compare the cumulative PNEN relapse-free survival after surgery between the 38 patients who underwent a major pancreatic surgery (PD + DP) and the 5 patients who underwent a minimal pancreatic surgery; relative plotted curves are reported in Figure 1. The log-rank test showed no significant difference between these two groups of surgeries (*p* value = 0.1189).

Of all the 43 patients, we reported only one PNEN-related death (2.3%), occurring at 61 years of age for multiple liver metastases from gastrinoma, diagnosed two years after the initial PD in which we had found and removed one duodenal gastrinoma, one gastrin-secreting lymph node metastasis, one gastrin-secreting liver metastasis, and nine NF-PNENs in the pancreas head. The recurrent liver metastases were unsuccessfully treated via radiofrequency liver ablation.

Post-operative outcomes in the two groups of patients treated with major or minimal pancreatic surgeries, expressed as the mean ± SD, median, and range of values, or as a percentage, are reported in Table 2.

## 4. Discussion

Despite increasingly early diagnosis and the improvement in surgical techniques, metastasized PNENs still represent the leading cause of death in MEN1 patients. NF-PNENs, which represent the most common form of these pancreatic tumors in MEN1 patients, may have an aggressive clinical behavior that appears related to their dimension, being about 30% for tumors of 2–3 cm and more than 50% for tumors greater than 3 cm. However, even if the majority of small PNENs (around 1 cm in diameter) present an indolent behavior, with no or very slow growth over the years, they are not devoid of malignant potential, and, when they become malignant, growth can occur in an accelerated and unpredictable manner.

The timing and extent of pancreatic surgery in MEN1 patients still remain matters of debate. Current ENETS guidelines suggest the use of circumscribed pancreatic surgery (enucleation, limited resection) in patients with a solitary PNEN identified via EUS, CT scan, or MRI [22]. This approach is reasonable for sporadic PNENs that almost always occur as a single tumor, while MEN1 patients are affected, in the great majority of cases, by multiple, often small, concurrent F-PNENs and/or NF-PNENs, scattered through the endocrine pancreas and the duodenal wall, which may be missed during conventional imaging and are recognized only during the surgical intervention [23]. Moreover, even if the most applied criterion is to operate MEN1-associated PNENs when these tumors have a size between 1 and 2 cm and/or manifest rapid growth (over 5 mm of diameter growth in the last year) [9,14,24], at the time of surgery, the presence of a malignant DP-NEN is found in about one third to half of patients (35–54%) [8,9,14,25]. In the present study, metastatic tumors were found in 27.9% of the 43 patients. Most of the removed metastases involved the peri-pancreatic lymph nodes and derived from small (<1 cm in diameter) multiple duodenal gastrinomas; gastrin-positive lymph node metastases were excised in 42.9% of our 21 patients operated for gastrinoma. In our series, only one case of NF-PNEN presented a lymph node metastasis. This patient had two NF-PNENs, both larger than 2 cm. Two insulin-positive lymph node metastases were removed in a patient with two primary macro-insulinomas (1.8 and 2.0 cm). Liver metastases were found in only two patients (4.7%), both operated via PD, and were related, respectively, to a duodenal gastrinoma and to a duodenal gastrinoma associated with a pancreatic VIPoma. Despite the fact that almost one third of our MEN1 patients had metastatic DP-NENs at the time of surgery, the metastatic progression or local recurrence of tumors at these advanced stages were very low (2/12; 16.7%). We reported only one patient who died of gastrinoma liver metastases that presumably were already present at time of the first surgery, but too small to be identified during the intervention. The overall survival during the study follow-up was 97.7%. 

In our experience, the most rational approach to prevent malignant progression and to increase the post-operative PNEN relapse-free survival time is a major partial pancreatic resection, aiming to remove the most affected part of the pancreas or the part presenting with larger tumors, associated with enucleation(s) or minimally pancreatic resection of all tumors greater than 1 cm, located in the remnant pancreas. In the presence of hypergastrinism, the preferred type of surgery was PD, since the great majority of MEN1 gastrinomas are located in the duodenal wall [25]. A major surgical approach was the one more commonly applied interventions in our series of MEN1 patients. Indeed, given the genetic nature of the MEN1 syndrome that predisposes patients to a very high risk of PNEN relapses after surgery, the performance of major surgical procedures, such as PD and DP, could reduce the risk of post-surgical tumor relapse, lengthen the post-operative PNEN relapse-free survival time, and delay/avoid the need for pancreatic reinterventions. Previous studies, performed by surgical centers with a wide experience in MEN1 DP-NENs, found the post-operative recurrence of PNENs to be variable between 35% and 79%, with a reoperation rate between 16% and 38% [8,9,12,14,26]. In our study, patients operated for PNENs via major pancreas resections, following the criterion of resecting the most affected part of the pancreas in association with the enucleation of tumors over 1 cm in the remnant pancreas and the removal of duodenal gastrinomas, manifested tumor relapse in 50.0% of cases in a median post-operative follow-up of 12 years (range: 2–26 years). 

Surgical reintervention for recurrences was necessary only in 5.3% of cases. The majority of relapsed patients had recurrences less than 1 cm in diameter. Only in one patient, the diameter of two recurrent NF-PNENs was greater than 2 cm, requiring a DP reintervention. The other reoperated patient was primarily submitted to this second surgery for the removal of an ectopic gastrinoma of the biliary tree, which was causing a medically unsolvable persistence of hypergastrinism. During the surgery, an NF-PNEN of 0.9 cm in diameter was also enucleated from the pancreas body. This specific case had been also reported in a previous publication [27]. No newly developed metastatic tumors were reported in all PD- and DP-operated patients. Our data confirmed previously published surgical experience [13] regarding the good effectiveness of pancreatic major surgeries in preventing tumoral progression.

Conversely, single-tumor enucleation and minimal pancreatic resection are usually accompanied by a higher percentage of persistence of endocrine syndromes and/or recurrence/relapse of PNENs. Our series presented only a limited number of minimal pancreas surgeries. No persistent endocrine syndrome was observed in all the five operated cases. One patient (20.0%) had a recurrence of endocrine syndrome 14 years after surgery, and two (40.0%) had relapses for NF-PNENs. Reoperation was performed in two (40.0%) of the recurrent cases. 

Considering the numerous variables that could influence the neoplastic progression of PNENs in MEN1, a clear answer of which is the best surgical strategy could be assessed via a prospective and randomized study, performed on MEN1 patients with PNENs between 1 and 2 cm, through evaluating tumoral outcomes after prophylactic surgery in one group of patients in comparison to a group of cases being only under surveillance. To date, such a study has never been planned. However, some authors have prospectively evaluated the risk of tumor progression in patients having non-functioning tumors less than 2 cm in diameter and not undergoing surgery [14,17], finding that the majority of these tumors remained stable or were growing very slowly. Conversely, tumors between 2 and 3 cm could metastasize very frequently, mostly in patients who did not undergo surgery [16].

Both major resections of the proximal or distal pancreas have a high risk of post-operative morbidity and mortality. Post-operative early complications were reported in every case series concerning major pancreatic resections performed for the MEN1 syndrome, varying from 26% to 63% of cases [7,13,19,28]. Grade III or higher Clavien–Dindo complications were observed in 63% of the 27 PD-operated cases of the Dutch MEN1 Study Group [19], while grade B or C post-operative pancreatic complications, classified according to the criteria of the International Study Group of Pancreatic Surgery (ISGPS), occurred in 26% of the 31 PD-treated cases of the Groupe d’étude des Tumeurs Endocrines (GTE) [28]. Also, DP is followed by frequent severe complications [13,19]. Clavien–Dindo grade III-IV complications, mainly consisting of grade B/C POPF or PPH, were observed in 31% of 36 MEN1 patients, from the Dutch MEN1 study group database, DP-operated for NF-PNENs [16]. However, a reliable evaluation of the risk of post-operative complications in these two major pancreatic surgeries, performed in the same surgical center, is still lacking, since the number of distal resections is usually far greater than that of proximal resections. Interestingly, despite being a more aggressive surgery, in our series, PD was not accompanied by more frequent post-operative complications; only one grade IV Clavien–Dindo complication was reported in our 19 PD-operated cases. We reported a higher number of patients presenting a post-operative complication and higher frequencies of POPF grades B and C PPH and PPAP in DP-operated patients; 3 grade III Clavien–Dindo complications were reported in our 19 DP-operated cases. We reported no post-operative early complication in all the five cases operated via minimal pancreatic resection or single-tumor enucleation.

The quality of life of MEN1 patients who are submitted to major pancreatic resections can be negatively affected by endocrine and/or exocrine insufficiency of the pancreas.

DM is a well-known sequela of major pancreatic resections, with a post-operative incidence that is strictly related to the extent of tissue removal, being, respectively 34%, 53% and 73% when <25%, 25–50%, or >50% of the pancreatic volume is removed [29]. Post-operative DM in MEN1 patients ranges from 22% to 50% after PD [9,28] and from 27% to 30% after DP [9,19]. In our study, DM arose in 37.2% of operated patients, more frequently after PD (52.6%), than after DP (26.3%) or minimal pancreatic surgeries (20.0%). The need to remove part of the left pancreas in addition to pancreas head is probably at the basis of what we observed in our PD-operated patients, in which DM prevalence was higher of that found in other similar studies [9,19]. This particularly high percentage of DM could be related to the increased insulin resistance, higher prevalence of impaired fasting glucose tolerance, and increased prevalence of DM often observed in MEN1 individuals than in controls [30,31]. Differentially from DM types I and II, pancreatogenic DM is well tolerated, being fairly sensitive to insulin administration, and rarely develops ketoacidosis or severe hyperglycemia or hypoglycemia [32], as confirmed also by our study, in which, in all patients manifesting post-operative DM, the hormonal impairment resulted to be well controlled through insulin supplementation, with minimal affection on the quality of life of patients.

Exocrine insufficiency of the pancreas is the other common sequela of major pancreatic resections, requiring pancreatic enzyme replacement after surgery. However, it is still a matter of discussion whether the reduced activity of pancreatic enzymes is a consequence of reduced exocrine function of the resected gland or due to a maldigestion caused by duodenum removal and modified intestinal transit [33]. Our study showed the occurrence of EPI, needing life-long pancreatic enzyme supplementation, to be significantly higher after PD (78.9%) than after DP (36.8%), confirming data previously reported by van Beek et al. [19], and suggesting the role of duodenum resection in increasing the occurrence of this malfunction. EPI occurred in one (20.0%) of the five patients who underwent a minimal pancreatic surgery, the same one who also developed DM, after resection of the pancreas tail and single enucleation in the pancreas head. Pancreatic enzyme supplementation was effective and well tolerated in all our treated patients with EPI, without significantly impacting their general quality of life.

## 5. Conclusions

In our opinion, and according to our long-term surgical experience and results from our operated series of MEN1 cases, in MEN1 patients, major pancreatic resections should be preferred in the care of both F-PNENs or NF-PNENs, especially in the presence of multiple tumors, including tumors less than 1 cm, scattered throughout the endocrine pancreas, for both the resolution of hormonal syndromes and the control of tumor growth, progression, and metastatic spread. The decision between PD or DP should be based on the distribution of PNENs, in terms of sites with a higher number of tumors or with the greatest neoplasms. In the presence of gastrinoma(s), PD should be preferred. The prevalence of post-operative sequelae, both for DM and EPI, is higher than for minimal pancreatic surgeries, but they appeared to be well managed through systemic therapies. 

Minimal pancreatic surgeries could be chosen and employed in specific cases, in which, at the time of surgery, the effect of the tumors on the pancreas still appeared limited, and tumor(s) can be resected through preserving a larger portion of the gland.

In our patients, minimal pancreatic resections showed a PNEN relapse-free survival time not significantly different from that of patients who underwent a major pancreatic resection. However, the Kaplan–Meier survival analysis was based on very few patients in the minimal surgery group (five patients only), and, thus, our result needs to be confirmed in a higher number of cases. Moreover, minimal pancreas resections were all employed in patients with a less tumor-affected endocrine pancreas and a lower number of tumors, without lymph node and distant metastasis, and initially referred to a less aggressive surgery because of a presumed more favorable prognosis, all these constituting selection bias that could have influenced the result of the Kaplan–Meier survival analysis.

## Figures and Tables

**Table 1:** Surgical characteristics in the two groups of patients treated with major or minimal pancreatic surgeries.

Parameter	Major Pancreas Resections (n = 38) PD (n = 19) DP (n = 19)	Minimal Pancreatic Surgeries (n = 5)	*p* Value
Age at MEN1 diagnosis (years) Mean ± SD Median (range)	Total: 31.4 ± 10.1 29.5 (15–57) PD: 35.7 ± 10.5 32.0 (21–57) DP: 27.1 ± 7.8 27.0 (15–48)	27.4 ± 10.2 24.0 (15–38)	0.41
Age at PNEN diagnosis (years) Mean ± SD Median (range)	Total: 35.5 ± 11.1 34.5 (17–63) PD: 38.3 ± 11.0 37.0 (25–59) DP: 32.7 ± 10.8 31.0 (17–63)	31.1 ± 11.6 28.0 (17–43)	0.40
Age at pancreas surgery (years) Mean ± SD Median (range)	Total: 37.1 ± 11.5 35.5 (17–63) PD: 40.4 ± 11.5 37.0 (27–60) DP: 33.7 ± 10.7 32.0 (17–63)	31.6 ± 11.7 29.0 (17–43)	0.27
Time between instrumental diagnosis of PNEN and surgery (years) Mean ± SD Median (range)	Total: 1.6 ± 3.0 1.0 (0–18) PD: 2.2 ± 4.0 1.0 (0–18) DP: 0.9 ± 1.3 1.0 (0–5)	0.6 ± 0.5 1.0 (0–1)	0.49
Post-operative follow-up (years) Mean ± SD Median (range)	Total: 12.1 ± 7.0 11.5 (2–26) PD: 12.2 ± 6.8 10 (3–25) DP: 12.0 ± 7.3 13 (2–26)	17.7 ± 6.8 17 (9.5–26)	Non applicable
Enucleation/resection in the preserved pancreas (n. and % of cases)	Total: 10/38 (26.3%) PD: 6/19 (36.8%) DP: 4/19 (21.0%)	1/5 (20.0%)	1
Liver resection (n. and % of cases)	Total: 4/38 (10.5%) PD: 4/19 (21.0%) DP: 0/19 (0%)	2/5 (40.0%)	0.14
Removed F-PNENs (%) - Gastrinoma (n = 21) - Insulinoma (n = 17) - VIPoma (n = 3) - Glucagonoma (n = 1)	Total: 19/38 (50%) PD: 16 (76.2%) DP: 3 * (14.3%) Total: 14/38 (36.8%) PD: 3 (17.6%) DP: 11 (64.7%) Total: 3/38 (7.9%) PD: 1 (33.3%) DP: 2 (66.7%) Total: 0 (0%) PD: 0 (0%) DP: 0 (0%)	2 (9.5%) 3 (17.6%) 0 (0%) 1 (100%)	1 0.37 1 0.12
Operated patients with NF-PNEN (n. and % of cases)	Total: 37/38 (97.4%) PD: 18/19 (94.7%) DP: 19/19 (100%)	2/5 (40.0%)	0.003
Number of removed NF-PNENs Mean ± SD Median (range)	Total: 11 ± 13 5 (0–60) PD 11 ± 11 8 (0–37) DP: 11 ± 15 3 (1–60)	8 ± 15 0 (0–35)	0.63
Diameter of the major removed PNEN (cm) Mean ± SD Median	Total: 2.2 ± 1.0 2.0 (0.1–5.0) PD: 1.9 ± 1.0 2.0 (0.1–4.0) DP: 2.4 ± 0.9 2.2 (1.4–5.0)	1.7 ± 0.9 1.4 (0.8–3.0)	0.48
Lymph node metastases (n. and % of cases)	Total: 11/38 (28.9%) PD: 11/19 ^#^ (57.9%) DP: 0/19 (0%)	0/5 (0%)	0.31
Liver metastases (n. and % of cases)	Total: 2/38 (5.3%) PD: 2/19 (10.5%) DP: 0/19 (0%)	0/5 (0%)	1

PD = pancreaticoduodenectomy; DP = distal pancreatectomy; PNEN = pancreas neuroendocrine tumor; F-PNEN = functioning pancreas neuroendocrine tumor; NF-PNEN = non-functioning pancreas neuroendocrine tumor; SD = standard deviation. * One case was a pancreatic gastrinoma and two cases were duodenal gastrinomas removed via duodenal enucleation associated with DP. ^#^ Nine cases were lymph node metastases positive to gastrin, one case was a lymph node metastasis positive to insulin in a patient with an insulinoma in the pancreas tail removed via enucleation associated with PD, and one case was a non-functioning lymph node metastasis in a patient with two NF-PNENs larger than 2 cm (2.3 and 4.0 cm). Parameters that showed a significant difference (*p* value < 0.05) between the two groups of patients treated with major or minimal pancreatic surgeries are highlighted in bold.

**Table 2:** Post-operative outcomes in the two groups of patients treated with major or minimal pancreatic surgeries.

Parameter	Major Pancreatic Resections (n = 38) PD (n = 19) DP (n = 19)	Minimal Pancreatic Surgeries (n = 5)	*p* Value
Grades III and IV Clavien–Dindo post-operative early complications (n. and % of cases)	Total: 4/38 (10.5%) PD: 1/19 (5.3%) DP: 3/19 (15.8%)	0 (0%)	1
Post-operative mortality (n. and % of cases)	0/38 (0%)	0/5 (0%)	Non applicable
Post-operative diabetes mellitus (n. and % of cases)	Total: 15/38 (39.5%) PD: 10/19 (52.6%) DP: 5/19 (26.3%)	1/5 (20.0%)	0.64
Post-operative chronic insufficiency of the exocrine pancreas (n. and % of cases)	Total: 22/38 (57.9%) PD: 15/19 (78.9%) DP: 7/19 (36.8%)	1/5 (20.0%)	0.17
Post-operative persistence of endocrine syndromes (n. and % of cases)	Total: 5/38 (13.2%) PD: 3/19 (15.8%) DP: 2/19 (10.5%)	0/5 (0%)	1
Post-operative relapse of F-PNEN(s) (n. and % of cases)	Total: 6/38 (15.8%) PD: 5/19 (26.3%) DP: 1/19 (5.3%)	1/5 (20.0%)	1
Post-operative relapse of NF-PNEN(s) (n. and % of cases)	Total: 15/38 (39.5%) PD: 5/19 (26.3%) DP: 10/19 (52.6%)	2/5 (40%)	1
PNEN relapse-free survival time after surgery (years) Mean ± SD Median (range)	Total: 9.4 ± 6.5 10 (0.5–25) PD: 8.7 ± 6.6 10 (1–25) DP: 10.2 ± 6.5 11 (0.5–20)	8.7 ± 5.3 9.5 (1–14)	0.65
Pancreas surgical reintervention(s) for persistence/relapse (n. and % of cases)	Total: 2/38 (5.3%) PD: 2/19 (10.5%) DP: 0/19 (0%)	2/5 (40.0%)	0.06

PNEN = pancreas neuroendocrine tumor; F-PNEN = functioning pancreas neuroendocrine tumor; NF-PNEN = non-functioning pancreas neuroendocrine tumor; SD = standard deviation.

## Data Availability

The dataset used and analyzed during the current study are available from the corresponding author on reasonable request.
